# Strategies to Overcome Resistance Mechanisms in T-Cell Acute Lymphoblastic Leukemia

**DOI:** 10.3390/ijms20123021

**Published:** 2019-06-20

**Authors:** Elena Follini, Matteo Marchesini, Giovanni Roti

**Affiliations:** Department of Medicine and Surgery, University of Parma, 43126 Parma, Italy; elena.follini@yahoo.it (E.F.); matteo.marchesini@unipr.it (M.M.)

**Keywords:** T-ALL, resistance, chemotherapy, glucocorticoid, IL7R signaling, PI3K-AKT-mTOR inhibitors, NOTCH1 inhibitors, BET inhibitors, CDK4/6 inhibitors, BCL2 inhibitors, selective inhibitor of nuclear export (SINE), DSRP

## Abstract

Chemoresistance is a major cause of recurrence and death from T-cell acute lymphoblastic leukemia (T-ALL), both in adult and pediatric patients. In the majority of cases, drug-resistant disease is treated by selecting a combination of other drugs, without understanding the molecular mechanisms by which malignant cells escape chemotherapeutic treatments, even though a more detailed genomic characterization and the identification of actionable disease targets may enable informed decision of new agents to improve patient outcomes. In this work, we describe pathways of resistance to common chemotherapeutic agents including glucocorticoids and review the resistance mechanisms to targeted therapy such as IL7R, PI3K-AKT-mTOR, NOTCH1, BRD4/MYC, Cyclin D3: CDK4/CDK6, BCL2 inhibitors, and selective inhibitors of nuclear export (SINE). Finally, to overcome the limitations of the current trial-and-error method, we summarize the experiences of anti-cancer drug sensitivity resistance profiling (DSRP) approaches as a rapid and relevant strategy to infer drug activity and provide functional information to assist clinical decision one patient at a time.

## 1. Introduction

T-cell acute lymphoblastic leukemia (T-ALL) is an aggressive cancer arising from T-cell committed lymphoblasts, with a diffuse invasion of bone marrow and peripheral blood [1]. According to the National Comprehensive Cancer Network (NCCN) data repository, the overall incidence of ALL is 1.58 per 100,000 individuals per year, with approximately 5970 new cases and ~1400 deaths in the United States (US) (2017) [2]. T-ALL comprises about 15% of ALL in children and 25% in adults. In patients with T-ALL, this aggressive disease typically presents itself with high white blood cells count, large mediastinal mass, organomegaly, and central nervous system (CNS) involvement.

**Figure 1:**
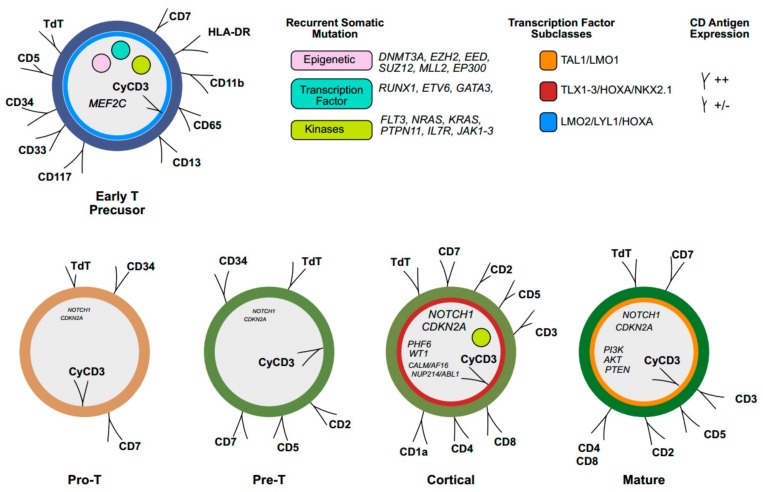
Immunophenotypic and molecular signatures of T-cell acute lymphoblastic leukemia (T-ALL). Early T precursors (ETP) show an immature phenotype with the expression of cluster of differentiation (CD) 34 and the myeloid markers (CD33, CD117, CD13, CD65, CD11b, HLA-DR), while typical antigens of T-cell are partially positive (+/−) or negative. Cytoplasmic CD3 (CyCD3) is a T-lineage marker, while the acquisition of other antigens such as terminal deoxynucleotidyl transferase (TdT), surface CD3 (sCD3), CD7, CD5, CD1, CD2, CD4 and CD8 varies during T-cell maturation from pro-T to pre-T, cortical and mature lymphocyte. ETP-cells display a genetic signature similar to myeloid stem cells, with a high frequency of mutations in genes involved in epigenetic regulation, kinase signaling, transcription factors, and *MEF2C*. T-ALL is divided into transcription factor subgroups (color coded circles): LYL1/LMO2, HOXA, TLX1, TLX3, NKX2-1 and TAL1/LMO1. *NOTCH1* and *CDKN2A* mutations rarely occur in immature T-cells while they are frequent in cortical subtype (the size of words shows frequency). Cortical-T ALL cells are more frequently associated with *PHF6*, *WT1*, *CALM-AF1*, *NUP214-ABL1*, and IL7R-signaling mutations. *PI3K*, *AKT*, and *PTEN* are frequently mutated in mature T-ALL cells.

T-ALL diagnosis rests on the integration of morpho-cytochemistry, flow cytometry, and genomics analysis. Because T-ALL lymphoblasts are morphologically indistinguishable from B-ALL (B-cell acute lymphoblastic leukemia), immunophenotypic characterization defines each stage of intrathymic differentiation by the expression pattern of cluster of differentiation (CD) antigens. T-ALL diagnosis relies on cytoplasmic and/or membrane CD3 positivity, while other T-cell antigens, such as terminal deoxynucleotidyl transferase (TdT), and CD7, CD2, CD5, CD1a, CD3, CD4, and CD8, are variably expressed during each step of development from pro-T to pre-T, cortical-T, and mature-T cells [3]. Immature thymocytes that maintain the ability to differentiate into both T-cell and myeloid lineage are characterized by the lack of CD8, CD4, CD1a, and CD5 (weak) expression, and the presence of at least one myeloid/stem cell antigen (CD34, CD33, CD13, CD11b, CD65, and/or HLA-DR) (Figure 1). This pattern identifies a subtype of T-cell leukemia, early T precursor (ETP) ALL, which accounts for 15% of pediatric and 35% of adult cases [4,5]. ETP-ALL is associated with an adverse outcome and poor prognosis with standard chemotherapy, both in pediatric and adult patients [6]. However, a recent analysis of the GRAAL (group for research on adult acute lymphoblastic leukemia) trial (2003 and 2005), showed that despite early chemotherapy resistance, ETP patients achieved similar five-year event-free survival (EFS) and overall survival (OS) rates compared to the non-ETP cohort [7]. Moreover, early intensification with hematopoietic stem cell transplantation in complete remission (CR) overcomes the negative impact of chemotherapy resistance in the ETP cohort [7].

Cytogenetic analysis has been the backbone to detect chromosomal abnormalities responsible for the activation of oncogenes or inactivation of tumor-suppressor genes involved in T-ALL development [8,9]. The incorporation of gene expression profiling into cytogenetic tools has provided new insights into T-ALL pathogenesis, while the T-ALL mutational landscape identified ~20 genes that are recurrently mutated [10]. These genes belong to one of the following ontological categories [11]: (1) transcription factors: *TAL1*, *TLX1*, *TLX3*, *HOXA*, *NKX2-1*, *LMO1-2/LYL1*; (2) Notch pathway: *NOTCH*-*MYC*-*FBXW7*; (3) cell-cycle regulation: *CDKN2A* and *CDK4/6* complex; (4) kinase signaling: *IL7R*, *JAK1/3*, *STAT5*, and *PI3K/AKT/mTOR*; (5) epigenetic factor: PRC2 complex, *KDM2A*; (6) non-coding RNAs: miRNA and lncRNA; (7) RNA metabolism/translation: *RPL5*, *RPL10*, *RPL11*, *RPL22*, CNOT3 complex, and EIF4A (Figure 1). Mutations do not occur randomly, but are grouped by clusters of frequently associated or mutually exclusive genes [10]. This suggests that cooperation of oncogenic events leads to the development of full-blown T-ALL, and similarly to other cancers; founder genetic events initiate the malignant process and subsequent acquisition of additional mutations or transcriptional and epigenetic aberrancies drive transformation and clonal evolution and eventually relapse [12,13,14]. Mutations in genes encoding for the apoptotic regulators (MDM2/p53) and for the epigenetic factors (PRC2) have been frequently found in relapsed T-ALL and may have a role in chemotherapy resistance [15,16,17]. Moreover, gain-of-function mutations of the cytosolic 5-nuceotidase 2 (*NT5C2*) have been typically associated with relapse.

The majority of patients with T-ALL are enrolled in clinical protocols based on the combination of different chemotherapeutic agents administered in sequential phases: induction, intensification, and maintenance [18]. The induction phase generally consists of steroids and high-dose chemotherapy with anthracyclines and vincristine in association with intrathecal methotrexate and cytarabine [19,20]. Given the evidence that depletion of extracellular asparagine is detrimental for lymphoblasts, the addition of l-asparaginase or PEG-asparaginase is highly recommended both in adult and pediatric protocols [21]. Once remission is achieved, intensification therapy with cyclophosphamide and high-dose cytarabine is considered the standard of care [22]. The maintenance phase generally lasts two years and is based on intermittent administration of the nucleotide analogue 6-mercaptopurine in association with pulse vincristine, prednisone, and central nervous system prophylaxis [23].

Allogenic bone marrow transplantation remains the only curative option for the majority of the relapsed/refractory (R/R) cases, since we do not have new drugs registered for this indication. In fact, nelarabine is the only drug (licensed in 2005) specifically for R/R T-ALL. In the registration study, the overall response rate to nelarabine was 41 to 46%, and the overall survival after one year accounted for 24–28% of responders [24,25], while pediatric trials showed a response rate varying from 14% to 55% of the cases [26,27]. These results indicate that nelarabine is insufficient as a salvage therapy, and indicate an urgent need for the development of novel agents. However, advances in genetic characterization have paved the way for the discovery of targeted therapies, with the combined goal to reduce the life-long off-target toxicities seen with the chemotherapeutic regimens, and the increase of complete remission (CR). Despite these efforts, resistance to standard chemotherapy, glucocorticoids, or new drugs remains the major hurdle to achieve meaningful progress in R/R T-ALL.

In this review, we will focus on resistance mechanisms to traditional chemotherapeutic approaches and to promising small-molecule inhibitors targeting pathway dependency, such as IL7R, PI3K-AKT-mTOR, NOTCH1, BRD4/MYC, Cyclin D3: CDK4/CDK6, BCL2, and selective inhibitors of nuclear export (SINE), and rationale combinations to enhance their activity (Table 1).

## 2. Mechanisms of Resistance to Standard Chemotherapy

As described above, T-ALL is an aggressive disease with a high risk of induction failure: up to 20% of children and 40% of adults relapse after intensive combination chemotherapy [60]. Relapsed T-ALL is associated with poor outcome with an estimated overall survival at five years of less than 7% [61]. Standard antracycline-based chemotherapy includes a combination of multiple agents, such as doxorubicine, vincristine, cyclophosphamide, cytarabine, and methotrexate. Several resistance mechanisms have been previously described including genetic, transcriptional, or epigenetic ways to escape from cell death [16,17].

For example, genetic disruption of nucleoside analogue metabolism is the major cause of resistance to 6 mercaptopurine (6-MP) [62]. The hypoxanthine-guanine phosphoribosyltransferase (HGPRT) converts 6-MP into thioinosine monophosphate (TIMP) by competing with the natural purine derivatives hypoxanthine and guanine, generally turning them into inosine monophosphate (IMP). TIMP inhibits several chemical reactions including the conversion of IMP to xanthosine monophosphate (XMP) and adenosine monophosphate (AMP), preventing the synthesis of purine bases and DNA assembly, causing cancer cellular death [63]. NT5C2 is a cytosolic nucleotidase that regulates intracellular nucleotide pool levels by exporting excess purine nucleotides out of the cell [62]. Among other substrates, NT5C2 can also dephosphorylate and inactivate the HGPRT metabolites 6-thioguanosine monophosphate (TGMP) and TIMP [62]. In 2013, Tzoneva et al. identified activating mutations in *NT5C2* in nearly 20% of R/R T-ALL. *NT5C2* mutations, including K359Q, R367Q, R238W, L375F, and D407A, lead to increased nucleotidase activity, conferring resistance to 6-MP and 6-thioguanine chemotherapy [16]. This hypothesis was confirmed both in T-ALL cell lines and in samples collected from R/R ALL patients showing a lack of cytotoxic responses in *NT5C2* mutated cases compared to wild-type [64]. Subsequent genetic and crystallographic studies revealed three classes of *NT5C2* mutations with different mechanisms of action. The type I mutations (K359Q and L375F) lock the allosterically activated helix A in a constitutively active configuration. The type II mutations (R39Q, R328W, R367Q, D407A, S408R, S445F, and R478S), which account for >95% of mutations, result in loss of the NT5C2 switch-off mechanism responsible for returning NT5C2 to its basal inactive state following activation. The type III mutations (Q523X) generate a truncated protein due to the loss of the C-terminal tail, impeding a switch toward an inactive protein state [65]. Collectively, these data identify three activating mechanisms by which *NT5C2* mutations increase nucleotidase activity, and pave the way for the development of *NT5C2* inhibitors to prevent and reverse purine analogue resistance in T-ALL [66].

Transcriptional imbalance of the murine double minute 2 (*MDM2*) gene is an established mechanism for resistance to several drugs [67]. *MDM2* negatively regulates the onco-suppressor protein p53 by promoting its ubiquitination [68]. Among other roles, p53 transcriptionally controls the expression of the ATP-binding cassette sub-family B member 1 (*ABCB1*), which encodes for the P-glycoprotein1 (P-gp) [69]. P-gp is an ATP-dependent membrane efflux pump involved in the pharmacokinetics of chemotherapy drugs, such as vincristine, anthracyclines, and glucocorticoids. Interestingly, p53 mutated or p53 deleted cancer cells display increased expression of the P-gp protein leading to chemoresistance [70].

Thus, it was hypothesized that MDM2 inhibitors could restore chemosensitivity in cancer cells. Nutlins are cis-imidazoline small molecule analogues, which inhibit the interaction between MDM2 and p53 [71]. Nutlin-3 was initially tested in neuroblastoma and rhabdomyosarcoma cell lines and was shown to inhibit the P-gp efflux pump [69] and synergize with the vinca alkaloid derivative vincristine. Nutlin-3 was further tested in acute myeloid leukemia (AML) cell lines and enhanced cytarabine and doxorubicine activity in p53 wild type cells [72]. In ALL, MDM2 overexpression is frequently observed [73,74,75], especially in T-ALL where cyclin-dependent kinase Inhibitor 2A (*CDKN2A*) disrupts p14, a negative regulator of MDM2 expression [76]. Moreover, preclinical trials showed the efficacy of Nutilin-3 analogue (RG7112 and MK-8242) treatment in mixed lineage leukemia (MLL) xenografted tumor models [77,78]. Interestingly, both studies showed that induction of remission was due to p53 reactivation and increased apoptosis. Based on these studies a multicenter phase I trial testing RG7112 is on-going in patients with haematological malignancies, including ALL [79].

In recent years, several examples of epigenetic drug resistance mechanisms have emerged in cancer therapeutics [80]. For, example, polycomb repressive complex 2 (PRC2) is a chromatin-modifying macromolecule, composed of the SUZ12 polycomb repressive complex 2 (SUZ12), enhancer of zeste homolog 2 (EZH2), and embryonic ectoderm development (EED) proteins. PRC2 is a methyltransferase that regulates transcriptional repression by controlling H3K27 methylation [81]. In T-ALL, loss of function PRC2 mutations enhance the oncogenic Notch transcriptional program [82] and repress apoptosis [17]. In fact, PRC2 loss induces chemoresistance to vincristine and doxorubicine, via transcriptional upregulation of the LIM domain transcription factor *CRIP2* and downstream upregulation of *TRAP1*, a mitochondrial chaperone that controls refolding of damaged proteins in the endoplasmic reticulum and prevents apoptosis [17].

In summary, these data suggest that resistance to chemotherapy may be due to genetic mechanisms or adaptive processes to escape from the selective pressure of cytotoxic agents.

## 3. Mechanisms of Resistance to Glucocorticoids

The therapeutic value of the adrenal glucocorticoids was discovered in 1949 [83], and from that time onwards, prednisone and dexamethasone became the backbone for the induction regimen in ALL. In fact, early blast-clearance during steroid prophase is an independent prognostic factor for complete remission and relapse-free survival (RFS) [84].

Glucocorticoid action is predominantly mediated through the glucocorticoid receptor (GR), NR3C1, which upon binding undergoes dimerization, and is translocated to the nucleus and interacts with a cognate DNA binding motif (GRE: glucocorticoid response element) [85]. Among other consequences, this interaction leads to transcription of the *BCL2L11* gene (BIM), and activation of a pro-apoptotic pathway in steroid-sensitive leukemic blasts.

While glucocorticoid receptor polymorphisms and haplotypes associated with resistance have been described [86,87,88,89], functional studies are lacking [90], supporting the hypothesis that resistance to steroids is mediated through altered signaling pathways rather than isolated genetic events. The majority of the studies focused on the following signals: IL7R and PI3K-AKT-mTOR.

### 3.1. IL7R Signaling Inhibitors

**Figure 2:**
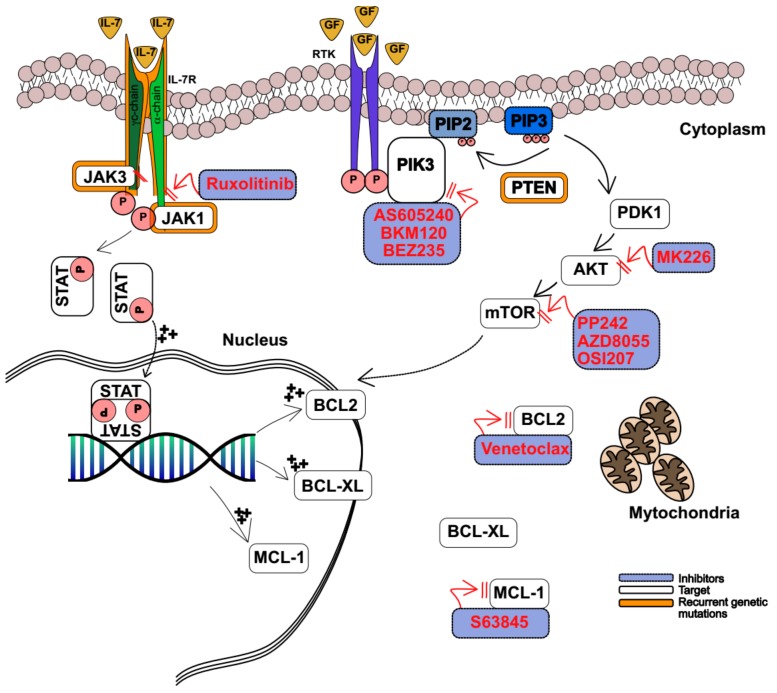
Kinase signalling pathway. In normal cells IL7 binds to its receptor IL7R. This interaction induces phosphorylation of Janus kinase 1 (JAK1) and JAK3 and activation of signal transducer and activation of transcription (STAT5) proteins. Phosphorylated STAT5, dimerizes and translocates into the nucleus and regulates the transcription of several genes, including the antiapoptotic BCL-2, BCL-XL, and MCL1. Growth factors bind to receptor tyrosine kinase (RTK), which trigger the activation of phosphatidylinositol-3 kinase (PI3K). PI3K phosphorylates phosphatidylinositol-3,4 bisphosphate (PIP2) into phosphatidylinositol-3,4, 5 trisphosphate (PIP3). PIP3 recruits the phosphoinositide-dependent kinase-1 (PDK-1) and the serine/threonine kinase (AKT) to the cell membrane, where they activate downstream effectors including the mammalian target of rapamycin (mTOR). Among its activities, mTOR induces the expression of the antiapoptotic protein MCL1. Phosphatase and tensin homolog (PTEN) are responsible for PIP3 dephosphorilation. In leukemic cells activating mutations in the IL7R-JAK-STAT pathway mediate steroid resistance, thus JAK1/2 inhibitors (ruxolitinib) could reverse glucocorticoid resistance. Activating mutations of *PI3K* and *AKT* are rare, while loss of PTEN is frequent in T-ALL. This leads to loss of negative control and persistent “on” status of AKT-mTOR signaling. Selective PI3K inhibitor (AS605240), dual PI3K-mTOR inhibitor (BEZ235), panPI3K inhibitor (BKM12), AKT inhibitor (MK2206), and mTOR inhibitors (PP242, AZD8055 and OSI027) act at different stages of this signaling pathway and may restore apoptosis in T-ALL cells. Hyperexpression (+++) of the antiapoptotic proteins BCL2 and MCL1 is counteracted by venetoclax or Mcl-1 inhibitors S63845, respectively. Black arrows show signal transduction. Red arrows show inhibition.

Interleukin 7 (IL7) is required for human T-cell development and homeostatic proliferation, through its interaction with the heterodimer IL7 receptor (IL7R) [91]. This interaction induces phosphorylation of JAK1 and JAK3, and subsequent activation of STAT5 proteins. Phosphorylated STAT5, dimerizes and then translocates into the nucleus, where it acts as a transcription regulator of several target genes, including the antiapoptotic BCL-2, BCL-XL, and MCL1 proteins [91]. Aberrant JAK-STAT signaling may result from the activation of a mutation in the IL7R pathway, which frequently occurs in the TLX, HOXA, and ETP T-ALL subgroups [92,93,94] (Figure 2). In addition, altered JAK expression derives from chromosome translocation t(9;12)(p24;p13), which generates the fusion of *ETV6-JAK2* [95]. The importance of IL7R signaling was demonstrated in a mouse model where Treanor et al. showed that hyperactive IL7R cooperates with *NOTCH1* mutations to induce T-ALL leukemia [96]. Interestingly, abrogation of *CDKN2A* in this model causes a leukemia phenotype similar to the ETP subgroup [96]. More recently, Tremblay et al. described an additional mechanism responsible for the aberrant expression of IL7R and activation of downstream signaling [97]. Here, the authors showed that inactivating mutations of dynamin 2 (*DNM2*), which is involved in IL7R clathrin-dependent endocytosis, leads to increased IL7R expression on the thymocyte surface [97]. An alternative way for T-ALL to alter IL7 signaling tone is by disrupting the negative feedback control mechanism mediated by Protein Tyrosine Phosphatase, Non-Receptor Type 2 (*PTPN2*). *PTPN2* is a protein phosphatase that dephosphorylates and inactivates JAK kinases. *PTPN2* loss-of function mutations occur in 7% of patients with T-ALL and, consequently, in these cases, T-ALL cells were more sensitive to cytokine stimulation, resulting in enhanced activation of JAK-STAT cytokine receptor pathways [98].

Mutations in IL7R signaling mediate steroid resistance [99]. Increased phosphorylation of JAK-STAT signaling in response to IL7 stimulation is common in glucocorticoid resistant T-ALL cells, and the addition of IL7 has been shown to induce steroid resistance in vitro [100]. Delgado-Martin et al. [28] demonstrated that T-ALL cells treated with 25 ng/mL of IL7 resist cell death induced by dexamethasone, compared to untreated cells. Interestingly, IL7 treated or untreated cells equally die upon standard chemotherapy treatment. In this setting, IL7 stimulation causes different levels of STAT5 phosphorylation, and T-ALL can be segregated into (a) highly responsive, (b) partially responsive, and (c) nonresponsive. Next, the authors asked whether STAT5 phosphorylation secondary to IL7 stimulation predicted response to steroids treatment, and the results showed that highly responsive T-ALL blasts failed to induce apoptosis upon dexamethasone treatment [28]. If phosphorylated STAT5 represents a biomarker of hyperactive IL7R-JAK-STAT signaling, an interesting hypothesis is whether JAK1/2 inhibition could reverse glucocorticoid resistance (Figure 2). Here, the authors demonstrated that T-ALL exposed to the combined treatment of dexamethasone and the JAK1/2 inhibitor ruxolitinib enhanced cell death in responder and partial responder patient derived xenograft (PDX) T-ALL in vitro. In fact, the combination of the two drugs alters the balance between pro-apoptotic and anti-apoptotic signals. Glucocorticoids down-regulate BCL2 and upregulate the pro-apoptotic BIM, while ruxolitinib inhibits IL7-induced expression of BCL2, resulting in enhanced apoptosis [28]. However, the lack of positive results in highly responsive T-ALL blasts suggests that mechanisms different from the IL7R-JAK-STAT axis cooperate with steroid resistance in IL7R mutant cases.

To further understand the genetic mechanisms that mediate steroid resistance, Meijerink et al. performed whole genome and targeted exome sequencing in 69 pediatric T-ALL samples [29]. As described in other works [10], mutations in *JAK1* and *KRAS*, were detected in 32% of patients and correlated with prednisolone resistance and reduced survival [29]. To functionally validate whether altered IL7R signaling drives steroid resistance, two steroid-sensitive cell lines (SUPT1 and P12-ICHIKAWA) were transduced with doxycycline-inducible constructs derived from a library encoding for *IL7R-JAK-RAS-AKT* mutations. Strikingly, expression of mutant *IL7R* RFCHP, mutant *JAK1* (*JAK1* R724H, *JAK1* T091A), wild-type *NRAS*, mutant *NRAS* G12D, and wild-type *AKT* conferred resistance to prednisolone in both cell lines [29]. Interestingly, NR3C1 expression and phosphorylation are equally expressed in steroid-sensitive and steroid-resistant cell lines, suggesting that this resistance mechanism is not primarily mediated by the glucocorticoid receptor. On the other hand, steroid-resistant T-ALL cell lines had greater activation levels of the PI3K-AKT-mTOR and RAS-MEK-ERK pathways, suggesting that these pathways may be responsible for the resistance mechanism.

### 3.2. PI3K-AKT-mTOR Inhibitors

The PI3K/AKT/mTOR signaling pathway regulates several cellular processes including cell cycle progression, cell metabolism, proliferation, and survival [101]. This pathway mediates receptor tyrosine kinase (RTK) activation, which upon ligand binding triggers the activation of phosphatidylinositol-3 kinase (PI3K) [102]. Next, PI3K phosphorylates phosphatidylinositol-3,4 bisphosphate (PIP2) into phosphatidylinositol-3,4,5 trisphosphate (PIP3). PIP3 recruits the phosphoinositide-dependent kinase-1 (PDK-1) and the serine/threonine kinase (AKT) to the cell membrane, where they subsequently activate downstream effectors including the mammalian target of rapamycin (mTOR) [102] (Figure 2).

PI3Ks have been divided into three classes according to their structural characteristics and substrate specificity. Of these, the most commonly studied are the class I enzymes (further divided into class A and B enzymes) that are activated directly by cell surface receptors [103]. Class IA PI3Ks are heterodimers consisting of a p110 catalytic subunit and a p85 regulatory subunit [104]. In mammals, there are three genes, *PIK3CA*, *PIK3CB*, and *PIK3CD*, encoding p110 catalytic isoforms: p110α, p110β, and p110δ, respectively. *PIK3CA* is among the most frequently mutated in several human malignancies including breast, colon, and prostate cancers [105].

The activation of PI3K-AKT-mTOR controls several T-cell physiological processes, such as T-cell differentiation, which requires the combined activities of PI3Kγ and δ [101,106]. In T-ALL, mutations of *PI3K* or *AKT* are sporadic (2%) [107], while the aberrant activation of this pathway is common. In fact, mutations and/or deletions of the phosphatase and tensin homolog (*PTEN*) gene occur in 15% of T-ALL cases [107,108,109]. Among other roles, *PTEN* is responsible for PIP3 dephosphorylation; thus, loss of the *PTEN* tumor-suppressor role determines a persistent “on” status of the AKT-mTOR signaling (Figure 1). Because several glucocorticoid resistance pathways merge on AKT-mTOR signaling, PIK3 modulation is expected to overcome resistance in T-ALL. For example, AS605240, a selective PI3Kγ inhibitor, was assessed in several T-ALL cancer cell lines (CCRF-CEM, HPB-ALL, JURKAT, MOLT-4, PI2-ICHIKAWA, ALL-SIL, and TALL-1), and in lymphoblasts derived from patients with T-ALL [30]. Global transcriptional analysis of cells treated with AS605240 revealed a suppression of *MYC* oncogene gene targets and gene set analysis (GSEA) enriched for signatures controlling energy metabolism, global transcription, and biosynthesis of cellular structure [30]. Combined treatment with AS605240 and prednisolone resulted in a synergist cytotoxic effect in several T-ALL cell lines in vitro, while the combination of AS605240 with dexamethasone prevented leukemia progression in PDX T-ALL NOD/SCID models [30]. Collectively, these data suggest that the PI3Kγ inhibitor may enhance steroid responses in T-ALL.

While selective molecules are in principal ideal to minimize off–target effects, pan-PI3K inhibition showed encouraging results in T-ALL models. For example, NVP-BKM120 (buparlisib), an oral pan-class I PI3K inhibitor belonging to the 2,6-dimorpholino pyrimidine derivative family, was shown to inhibit T-ALL proliferation while dephosphorylating AKT and S6RP in vitro. Moreover, in vivo administration of NVP-BKM120 in a PDX model of human T-ALL significantly delayed tumor growth, prolonged survival time, and synergized with chemotherapeutic agents currently used for treating T-ALL patients [35]. NVP-BKM120 is currently in clinical investigation for patients with advanced leukemia in a phase I clinical trial NCT01396499 [36].

An additional strategy to modulate steroid resistance is by inhibiting the PI3K effector AKT-mTOR [31] (Figure 2). MK2206 is a dihydrochloride, orally active, allosteric AKT inhibitor developed by Merck. Piovan et al. demonstrated that MK2206 inhibits AKT signaling, causing a reduction of phosphorylated mTOR in T-ALL. Next, they showed that MK2206 dephosphorylates the glucocorticoid receptor NR3C1 in position S134, enabling its translocation into the nucleus and restoring steroid sensitivity in CCRF-CEM and MOLT3, two PTEN null, glucocorticoid-resistant T-ALL cell lines [31]. In pre-clinical translational studies xenograft models of glucocorticoid-resistant T-ALL were treated with dimethyl sulphoxide (DMSO), dexamethasone, MK2206, or a combination. Mice treated with MK2206 plus dexamethasone showed a significant reduction of tumor burden, while tumor growth was similar in the other study, suggesting that AKT modulation may control glucocorticoid resistant cells in T-ALL [31].

In the last decade, several dual inhibitors have been developed for cancer treatment [110]. An example is the dual PI3K-mTOR inhibitor NVP-BEZ235 (Dactolisib), which simultaneously binds to the ATP-binding pocket of PI3K and mTOR (Figure 2). Pre-clinical studies demonstrated that NVP-BEZ235 triggered a G0/G1 cell-cycle arrest and caused caspase-mediated apoptosis in T-ALL [32]. In addition, NVP-BEZ235 synergizes with dexamethasone (combination index, which defines synergism (CI < 1), additive effect (CI = 1), and antagonism (CI > 1), equal to < 3) in T-ALL cells isolated from pediatric patients compared to T-ALL cells treated with single agents [33]. Finally, the authors showed that NVP-BEZ235 restores steroid mediated apoptosis by facilitating BIM pro-apoptotic signals and suppressing MCL1 anti-apoptotic activity [33]. Because of these pro-apoptotic properties, and additional studies showing that NVP-BEZ235 synergizes cytarabine and doxorubicine in T-ALL [34], dactolisib rapidly translated into a phase I clinical trial in adult patients with relapsed-refractory acute leukemia (NCT01756118).

In addition to dual inhibitors, several selective mTOR kinase modulators have been developed in the last two decades [111]. These small molecules target the mTOR catalytic subunit of the mTORC1 and mTORC2 complexes [111]. For example, PP242 (also known as Torkinib) is a potent, selective, and ATP-competitive inhibitor of mTOR, and it is among the most broadly used in preclinical models because of the demonstrated anti-proliferative and pro-apoptotic activity in several cancer models, including ALL [37]. Similarly, AZD8055, a selective mTORC1 and mTORC2 inhibitor, reduced MCL1 expression and induced tumor regression in vivo in different T-ALL disease models [38].

Few experiences assessed the role of mTORC1 and mTORC2 combined inhibition. For example, OSI-027, an inhibitor of the mTOR catalytic site, blocks the mTORC pathway by reducing 4EBP1 phosphorylation and triggering an anti-leukemia effect by decreasing *MYC* expression and increasing the expression of the pro-apoptotic BCL2 family member p53 upregulated mediator of apoptosis (*PUMA*). Furthermore, inhibition of mTORC2 resulted in Nuclear Factor-kB-mediated expression of the Early Growth Response 1 (*EGR1*) gene, which encodes a transcription factor that binds and trans-activates the *BCL2L11* locus, encoding the pro-apoptotic protein BIM [112].

The PI3K-AKT-mTOR axis may crosstalk with several signals including the RAS-MAPK-ERK pathway to engender a steroid resistant state in T-ALL. Recent studies demonstrated that RAS-MAPK-ERK signaling is aberrantly activated in 10–15% of T-ALL, with a slight prevalence for pediatric patients and for the ETP subtype, due to activating mutations of *KRAS*, *NRAS*, and *PTPN11* or inactivating mutations of *NF1* [12]. Thus, inhibition of this pathway is expected to suppress T-ALL growth. For example, CI1040 is a highly selective ATP non-competitive MEK1/2 inhibitor that prevents ERK phosphorylation, and subsequent signaling showed promising results in several disease models [113]. In SUPT1 and P12-ICHIKAWA T-ALL cells, CI1040 reduced downstream activation of mTOR and prevented BIM phosphorylation by ERK, restoring steroid sensitivity [29].

Taken together, these results suggest that the potent cytotoxic effects of inhibiting both PI3K-AKT-mTOR and RAS-MAPK-ERK (alone or in combination with chemotherapeutic drugs) may increase steroid responsiveness, and should be investigated further, since they may represent an effective treatment in a subset of aggressive T-ALL.

## 4. Emerging Approaches and Resistance Mechanisms

### 4.1. Notch Pathway Inhibitors

The Notch1 pathway plays a key role in several T-cell processes, including stem-cell self-renewal, proliferation, and thymic differentiation [114,115]. The active form of the NOTCH1 (ICN1, NICD) receptor results from a series of proteolytic cleavages from a full-length protein that traffics from the endoplasmic reticulum, to the surface of the cells, and finally back to the nucleus. The first cleavage is mediated by furin-like protease (S1) and is responsible for NOTCH-receptor maturation from the ER/Golgi to the cell membrane. The second cleavage is triggered by the ADAM metalloprotease (S2) and occurs in the NOTCH extracellular domain after ligand-receptor binding. The last cleavage is mediated by γ-secretase complex (S3) within the transmembrane domain and releases the intracellular form of NOTCH1 (ICN1), which translocates into the nucleus and activates gene expression. The presence of multiple processing steps makes the NOTCH-pathway very appealing for inhibitory-drug development [42,116,117,118,119]. Furthermore, the preponderance of oncogenic *NOTCH1* mutations in T-ALL (40% to 70% of childhood and adult T-ALL) [120,121] has driven the search for effective anti-Notch1 therapeutics. Because Notch activation relies on γ-secretase mediated proteolysis, gamma secretase inhibitors (GSIs) have entered in clinical trials for treatment of relapsing T-ALL. GSIs are widely known among neurologists because of their ability to block β-amyloid production in Alzheimer’s disease [122], but as anti T-ALL agents have experienced several roadblocks. In T-ALL GSIs induce cell cycle arrest rather than apoptosis [39], suggesting that as a single agent they may be insufficient to eradicate leukemia cells. In fact, only a few studies reported a complete response to GSI treatment [40]. However, several investigators demonstrated the benefit of combining GSIs with other agents in leukemia and solid tumors [39]. For example, glucocorticoids prevent GSI mediated gastro-intestinal toxicities [123], and in turn, GSIs enhance responses to steroids such as dexamethasone [124]. Furthermore, the combination of GSIs and dexamethasone in CUTTL1 T-ALL cells upregulates transcription of the glucocorticoid receptor (NR3C1), glucocorticoid-regulated genes, and the proapoptotic BIM proteins [125]. Further experiments demonstrated that loss of *HES1*, a transcriptional repressor controlled by NOTCH1 that binds to each of the three-glucocorticoid receptor promoters, upregulates glucocorticoid receptor expression and consequently re-sensitizes the cell to glucocorticoid therapy [124,125]. Thus, GSI mediated positive feedback transcriptional activation may revert a general mechanism of NR3C1 repression observed in T-ALL resistant cell lines [126,127].

In addition, several investigators described genetic mechanisms to explain resistance to Notch suppression in T-ALL [128]. First, T-ALL cells carrying *FBXW7* mutations present a delayed ICN1 degradation and stabilization of MYC proteins, engendering a GSI resistant state [129]. Secondly, loss of PTEN and the consequent activation of the PI3K pathway renders T-ALL cells less dependent on NOTCH1 signaling for their growth and proliferation, and thus GSI therapy turns out to be less effective [108].

Knoechel et al. identified an additional mechanism of tolerance to GSI therapy. In this work, the authors described a subpopulation of T-ALL cells called “persister” which were resistant to prolonged GSI treatment. Resistance to GSI was reversible after the drug’s withdrawal; hence, they speculated an epigenetic mechanism of drug resistance. Next, they performed a short hairpin RNA (shRNA) screen targeting genes involved in chromatin regulation. Among the top hits, which preferentially impaired the viability of “persister” cells while sparing the naïve population, they identified the BET (bromodomain and extra terminal domain) family BRD4. These T-ALL cells reactivate MYC expression via a distal group of BRD4-dependent enhancers which are independent from NOTCH1 signaling. Consistently “persister” cells were more sensitive to BRD4 inhibition (JQ1) in vitro, and combination therapy targeting “naïve” (GSI) and “persister” (JQ1) was significantly more effective in T-ALL xenotransplant models in vivo [40].

Despite the initial lukewarm results of GSI clinical trials in T-ALL, NOTCH1 remains one of the most desirable targets in this disease, and several efforts are on-going to improve selectivity (NOTCH1 versus NOTCH2-4) [41] and reduce off-target effects (mutated NOTCH1 versus wild type) [42].

### 4.2. BET Inhibitors

Acetylation of the N-terminal histone tails represents a post-translational modification responsible for open-chromatin structure that facilitates euchromatin transcription. The bromodomains (BRDs) are conserved protein modules of 110 amino acids that recognize and dock themselves to *N*-acetylated lysine residues [130]. BRD proteins include histone transferases (HATs) and HAT associated proteins, methyltransferases, helicases, chromatin remodelers, transcriptional co-activators, and BRD extra terminal proteins (BETs) [130,131]. The BET proteins comprise a family of four proteins: BRD2, BRD3, BRD4, and BRDT, which use two tandem BRD domains to recognize *N*-acetylated lysine residues to recruit transcription factors, both in physiological and disease processes such as inflammation and cancer [131,132].

The direct involvement of a BET family member in human cancer was originally defined in 2001 [133,134]. French et al. described an aggressive thoracic squamous cell tumor, defined ad midline carcinoma, carrying a t(15;19) that fuses *BRD4* to *NUT*. A further advancement in the field was due to the open-source approach of Bradner’s laboratory, which developed and distributed a potent BET bromodomain inhibitor, JQ1. JQ1 is a thieno-triazolo-1,4-diazepine that binds selectively to the acetyl lysine pocket of the BRD module and was shown to inhibit cancer cell proliferation in several tumor models, including the BRD4-NUT carcinoma, generally by inhibiting Myc signaling [135].

In several hematological malignancies, *MYC* is transcriptionally regulated by super-enhancers, which are broad areas of active open chromatin marked by H3K27 acetylation. Because BRD4 densely occupies super enhancers, its inhibition reduces *MYC* transcription causing an anti-tumor effect [136]. Two studies demonstrated that acute myeloid leukemia (AML) and multiple myeloma depend on the BRD4-MYC axis, and that pharmacological modulation of JQ1 inhibited cancer cell proliferation in vitro and in vivo [137,138].

The potential of BET inhibitors in T-ALL was described in several studies [43,44,139]. King et al. demonstrated that treatment with BET inhibitors repressed MYC protein expression and arrested leukemia cell growth in T-ALL cell lines, a result consistent with the one obtained by the genetic abrogation of *MYC* and *BRD4* in NOTCH1-positive T-ALL [140]. Next, they generated a mouse-model carrying a *FBXW7*-R465C mutation. Because *FBXW7* mutations affect the stability of NOTCH1 and MYC proteins, this model was characterized by a marked increase of leukemia initiating cells (LIC), due to a greater MYC protein concentration. Furthermore, the authors demonstrated that the JQ1 derivative (CPI203) inhibited T-ALL growth in mouse models of either FBXW7-mutated or wild type T-ALL, suggesting that FBXW7-mediated GSI resistance may be overcome by CPI203 treatment [139]. Interestingly, JQ1 and I-BET151 inhibit the proliferation of ETP T-ALL PRC2 inactivated cells due to *EZH2* and *RUNX1* mutations both in vitro and in vivo [141].

In a subsequent study, Roderick et al. confirmed the role of MYC in LIC maintenance in T-ALL [43]. First, they demonstrated that JQ1 suppresses cell proliferation in a collection of human T-ALL cell lines, in T-ALL lymphoblasts collected from pediatric patients with R/R T-ALL. Next, they established a *MYC* repressible NOTCH1 mutant TAL1/LMO2 T-ALL mouse model, and demonstrated that *MYC* abrogation depletes LIC and consequently prolongs survival in treated mice. Consistently, JQ1 pharmacological inhibition reduced MYC expression in TAL/LMO2 T-ALL murine cell-lines, causing a degree of growth suppression greater than the one achieved with GSI [43].

In addition to the studies described above, Loosveld et al. completed a small molecule screening in eight T-ALL cell lines testing epigenetic modifiers or chemotherapeutic agents. Interestingly, T-ALLs were sensitive to JQ1 and histone deacetylase (HDAC) inhibitors, and both synergize with vincristine in vitro and in T-ALL PDX models in vivo. They demonstrated that MYC expression correlates with the degree of responses to JQ1, HDAC, and vincristine, emerging as a potential tumor biomarker of T-ALL [44].

In summary, these data suggest that BET bromodomain inhibition may represent a feasible strategy in R/R T-ALL, especially in cases with MYC overexpression.

### 4.3. CDK4/6 Inhibitors

**Figure 3:**
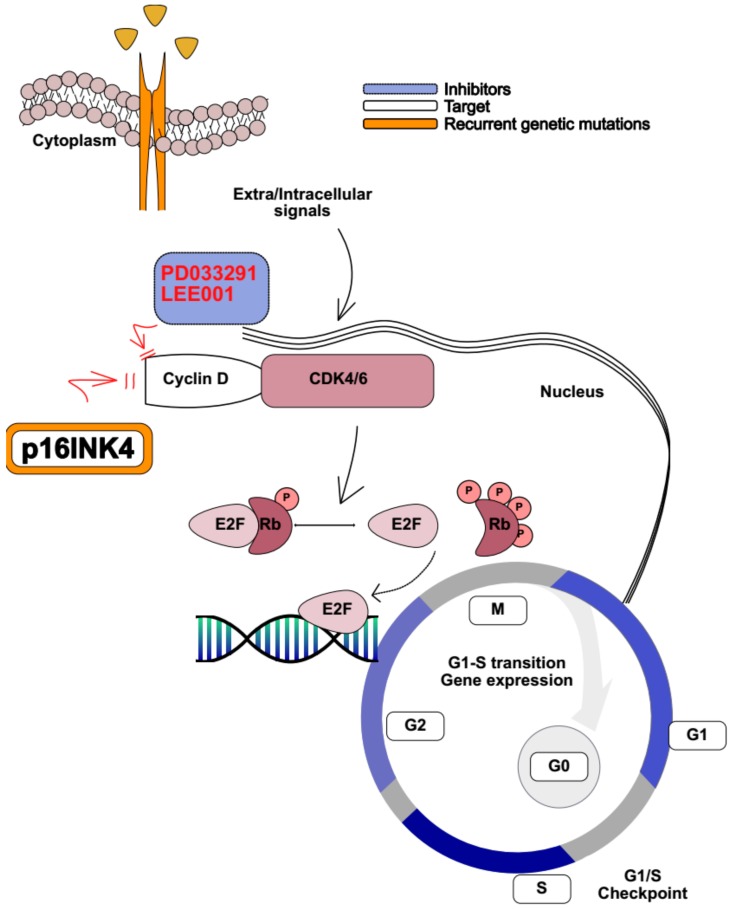
Cell cycle regulation. In normal cells p16INK4A, encoded by *CDKN2A*, inhibits the CDK4/6-Cyclin D complex by binding to it and preventing phosphorylation (P) of retinoblastoma (Rb), which holds its binding to E2 factor (E2F) family of transcription factors, switches gene transcription off, and inhibits G1 to S phase. In T-ALL cells, loss of CDKN2A causes loss of p16INK4A. CDK4/6-Cyclin D phosphorylates Rb, which releases E2F and promotes its binding to DNA, enabling gene expression and G1/S transition. Cyclin D/CDK4-6 inhibitors (PD033291 and LEE001) restore cell cycle regulation by binding to the CDK4-6/Cyclin D complex, mimicking p16INK4A function. Black arrows show signal transduction. Red arrows show inhibition.

Loss of *CDKN2A* is among the most frequently recurrent abnormality in T-ALL, where chromosome 9p deletions recur in 60–70% of cases [76] (Figure 3). *CDKN2A* encodes for the tumor suppressor genes *p16INK4A* (p16) and *p14ARF* (p14), which coordinate with cyclin D (D1, D2, and D3) during cell cycle progression [142]. Cyclin D variants interact with four cyclin-dependent kinases (CDK2, 4, 5, and 6). In proliferating cells, the cyclin D CDK4/6 complex partially phosphorylates retinoblastoma tumor suppressor protein (Rb), which becomes inactive and unable to bind the transcription factor E2F1 [143]. This signaling cascade leads to the expression of E2F1-dependent genes involved in cell-cycle progression, and in S phase transition [143]. Amplification of cyclin D genes and overexpression of their related proteins is a common feature of several human cancers [144]. For example, increased expression of cyclin D1 is observed in breast cancer [145] and increased expression of cyclin D2 in ovarian and testicular tumors [146], while cyclin D3 overexpression is a common feature of lymphoid malignancies [147]. In addition, D cyclins are required for tumor initiation, as mice lacking cyclin D1 are resistant to ErbB2-driven mammary adenocarcinomas [145,148], while cyclin D3 null animals are refractory to Notch1-driven T-ALL [147]. In addition to *CDKN2A* mutations, other genetic events disrupt cell cycle control in T-ALL. *CDKN1B*, located on 12p13.2, is deleted in 12% of T-ALL and causes cell-cycle arrest via p27 inhibition of cyclin E-CDK2 and cyclin D-CDK4 complexes [149].

To determine whether T-ALL cells depend on cyclin D for tumor maintenance, Choi et al. induced acute ablation of cyclin D3 in an ICN1 expressing T-ALL mouse model. A strong reduction in the number of leukemic cells, and consequently, a prolonged survival in cyclin D3 KO mice was observed [45]. They next tested whether a CDK4/6 inhibitor, PD0332991 (palbociclib), had a similar impact in T-ALL xenografts. Consistently treated animals displayed a survival advantage compared to the control [45]. Sawai et al. showed similar results [46]. In fact, PD0332991 administration efficiently suppressed T-ALL leukemia growth both in vitro and in vivo, by inducing apoptosis mediated by Rb (S807/811) phosphorylation, and increasing the expression of negative regulators of mitosis such as p27Kip1 CDKI [46]. Interestingly, PD0332991 treatment in vivo was tolerable, suggesting that cyclin D functions are redundant in many tissues, and another could replace the lack of one individual type. However, overexpression of cyclin D2 failed to rescue the loss of cyclin D3 in a T-ALL initiating model, indicating that cyclin D3 is required for tumor establishment in this disease.

Because the CDK4/6 inhibitor represents an ideal target in several human cancers, studies aimed to identify potential synergistic combinations [150,151]. Given that chemotherapy agents are active on highly proliferating cells and CDK4/6 inhibitors induce cell-cycle arrest, their combination would be predicted to be antagonistic. Indeed, Pikman et al. showed that the CDK4/6 inhibitor LEE001 (ribociclib) administered concomitantly or subsequently with methotrexate, mercaptopurine, doxorubicine, and L-asparaginase exerts an antagonistic effect in several T-ALL cell lines in vitro (Figure 3). On the other hand, LEE001 synergizes with glucocorticoids and the mTOR inhibitor everolimus both in vitro and in vivo, in T-ALL xenograft models established from MOLT4 and MOLT16 [47], suggesting that CDK4/6 inhibitors may emerge as a potential strategy in R/R T-ALL. Finally, ongoing early phase clinical trials are exploring LEE001 use in patients with R/R leukemia (NCT02310243, NCT03132454, NCT03472573, NCT03792256).

In summary, these results demonstrate that CDK4/6 inhibitors, alone or in association with other target therapies, may serve as promising small molecules with antiproliferative effects on several tumors, including T-ALL.

### 4.4. BCL2 Inhibitors

Altered expression of the pro-survival BCL-2 family proteins (BCL-2, BLC-XL, and MCL1) is a hallmark feature of human cancers, resulting in the activation of complex cellular strategies to evade apoptosis [152]. All family members share homology in at least one of the four BCL2-homology domains (BH1-4 domains) [153]. BCL2 and BCL-XL share homology in all four BH regions, while BIM, PUMA, BID, BAD, BIK, BMF, NOXA, and HRK, called BH3-only domain proteins, share homology only in the BH3 domain [154]. Thus, there is great interest in the development of drugs that mimic the action of BH3-only domain proapoptotic proteins by binding to one or more BLC2-like proteins and triggering the apoptotic program.

In the last 10 years, several BH3-mimetics have been developed and tested in different cancer models [152,155,156], including leukemias [157,158,159]. However, the lack of target selectivity against BCL-2, BCL-XL, and BCL-W limited the application of many of them [160]. For example, ABT-737 (navitoclax), an orally bioavailable derivative, was tested in a phase I clinical trial in adult patients with chronic lymphocytic leukemia (CLL). Despite encouraging results, the study revealed that ABT-737 induced thrombocytopenia due to the inhibition of BCL-XL, thus limiting its clinical development [161]. To overcome these limitations, Abbvie designed ABT-199 (venetoclax), an orally available small molecule selective for BCL-2 [162]. Venetoclax is approved by the FDA for patients diagnosed with CLL bearing a 17p deletion who have received at least one prior therapy [163] or in newly diagnosed AML in combination with azacitidine, decitabine, or low-dose cytarabine in adults >75 years who are not fit for induction chemotherapy [164]. Currently, several clinical trials are testing these molecules alone or in combination with other drugs in non-Hodgkin lymphoma (NCT02187861, NCT02956382, NCT02987400), multiple myeloma [165], and acute myeloid leukemia [157,158,159].

Several preclinical studies tested BH3-mimetics in ALL leukemia models [48,49,50]. For example, Alford et al. tested ABT-263 and ABT-199 in RS4;11 and NALM-6 B-ALL cell lines, and in 14 primary samples (12 adult and two pediatric), and showed that both compounds suppress leukemia proliferation [48]. Moreover, ABT-263 was tested in vivo in PDXs obtained from 31 pediatric patients with genetically defined T-ALL, B-cell precursor ALL, or MLL-rearranged ALL. ABT-263 showed in vivo efficacy with no apparent subtype specificity and induced regressions in 19 of 31 xenografts [51]. Complete molecular remission was achieved in all three T-ALL PDXs, and two partial remissions and 1 complete remission were obtained in ETP, indicating a privileged application in this setting of aggressive ALL [51]. To further investigate the potential use of ABT-199 in T-ALL, Peirs et al. analyzed the BCL2-expression level in 64 human T-ALL samples. Distinct T-ALL genetic subtypes showed different levels of *BCL2* expression. Immature, HOXA/TLX1/TLX3 and mature TAL1 T-ALL presented high, intermediate, and low levels of BCL2-expression, respectively. Immunophenotypic analysis confirmed this initial observation and showed that BCL2-expression correlates with the stage of blast differentiation arrest. Namely, CD34 positive cells present a greater BCL-2 level compared to CD4+ CD8+ cells, suggesting that more undifferentiated T-ALL leukemia cells express greater levels of BCL-2. Consistently, the immature, ETP-like, T-ALL cell line Loucy and its orthotopic mouse model displayed the greatest sensitivity toward ABT-199 treatment, similar to 17 primary ETP blasts and HOXA-positive PDXs [49].

In a parallel experience, Chongaille et al. developed a mitochondrial BH3 profiling assay and showed that ETP-like T-ALL cell lines and pediatric ETP lymphoblasts were more dependent on BCL-2 compared to other T-ALL subtypes that are generally more BCL-XL dependent. Consistent with this data, ETP cells and ETP-PDXs showed greater sensitivity to ABT-199, while T-ALL cells and PDXs not bearing ETP-features were more sensitive to treatment with ABT-263 [50]. In conclusion, together, these studies highlight that BCL-2 expression and response to BCL-2 suppression depend on T-ALL genetics and immunophenotype, which could be exploited as biomarkers for therapy.

The introduction of these new agents rapidly raises the question of resistance. The majority of the studies point to a transcriptional feedback mechanism to escape from BCL-2 dependency [166,167]. For example, the downregulation of BCL-2 reflects the loss of addiction to this protein, but the compensatory upregulation of the anti-apoptotic proteins not targeted by venetoclax (BCL-XL and MCL1) is observed in leukemia or cancer cell line venetoclax-treated cells [168]. Consequently, the combination therapy with standard chemotherapeutic agents [52] or small molecules may serve as a valid strategy to overcome venetoclax resistance and dose-related toxicity.

Recently, Li et al. developed a new BH3-mimetic selective for MCL1, S63845, which was shown to be active in the majority of T-ALL cell lines. Interestingly, in T-ALL expressing high levels of MCL1, S63845 reverted resistance to venetoclax, as shown by isobologram analysis of drugs synergy [53].

However, a solution may come from “old” drugs. The FDA approved proteasome inhibitor bortezomib downregulates MCL1 through the induction of NOXA, a protein associated with MCL1 degradation [169]. Bortezomib is largely used in clinical practice in multiple myeloma [170] and is currently being tested in preclinical studies in T-ALL [54], where it was shown to cause cellular death in a large panel of T-ALL cell lines at sub-nanomolar concentrations, and to suppress the expression of Notch and its target genes (*HES1*, *GATA3*, and *RUNX3*), as well as to hamper NF-Kb activity. As for other cancers, bortezomib synergizes with several other drugs, including chemotherapy and glucocorticoids. Interestingly, the combination of bortezomib and dexamethasone lead to almost complete remission of T-ALL xenografted tumors in vivo [54].

An additional mechanism of resistance to venetoclax, that can be relevant for T-ALL, has been observed in B cell malignancies. In fact, a venetoclax-treated resistant diffuse large B-cell lymphoma cell line (DLBCL) expresses a high level of phosphorylated AKT and suppresses PTEN. Interestingly, the dual selective PI3K inhibitor KA2237 restored the sensitivity of these cells, suggesting that overexpression of the RTK may contribute to resistant BH3-mimetics [55]. Consistently, the combined treatment of JAK2 inhibitor ruxolitinib and venetoclax reduced leukemia burden in an IL7R mutant T-ALL mouse model in vivo [56].

In conclusion, these experiences suggest that inhibition of the BCL-2 axis may become a valuable approach in T-ALL, especially in ETP-ALL, where the combination of venetoclax with other drugs may increase survival in patients with R/R T-ALL (Figure 2).

### 4.5. Selective Inhibitors of Nuclear Export

Selective inhibitors of nuclear export (SINE) are a drug family with a novel mechanism of action, which has been recently tested in solid tumors and leukemias [171]. Nucleo-cytoplasmic trafficking is critical for several cellular processes, including proliferation and survival [172]. Chromosome region maintenance1 (CRM1, also termed XPO1) is one of the seven exportins which mediate the transport of almost 200 proteins and mRNAs [173]. CRM1 is the only exportin involved in transport of tumor suppressor and growth regulatory proteins, such as p53, p21, p73, Rb1, APC, BCR-ABL, FOXO, and STAT3 [171]. Physiologically, these proteins are exported out of the nucleus, preventing them from acting in the absence of DNA damage. Conversely, in cancer cells, their transport in the cytoplasm by CRM1 inhibits their tumor suppressor activity, leading to tumorigenesis [171]. Mis-localization of tumor suppressor proteins, cell cycle regulators, and pro-apoptotic proteins is an emerging mechanism for chemoresistance [57]. Thus, the development of selective inhibitors of nuclear transport (SINE) could be a promising and alternative approach to treat cancers [58].

Several SINE have been developed; however, KPT-330 (selinexor), a potent, selective, and orally bioavailable CRM1 inhibitor, is the most used in preclinical and early clinical trials (NCT022112561, NCT02091245). Etchin et al. showed that KPT-330 induced apoptosis in 14 T-ALL cell-lines, and dramatically suppressed the MOLT-4 growth of cells engrafted into NOD-SCID-IL2Rg null (NSG) mice with no effect on normal hematopoiesis [59]. Although preliminary, these data suggest that SINE could be a valid and innovative approach to target T-ALL. Their association with small molecule inhibitors and their feasibility in early clinical trials are questions that need to be clarified further.

## 5. Preclinical Screening as a Strategy to Overcome Resistance

Although in recent decades, we welcomed the advent of targeted therapies in several cancers, nelarabine, a purine nucleoside antimetabolite, is the only FDA agent approved in the setting of R/R T-ALL. This is in part due to the lack of new agents for R/R T-ALL, and the need for alternatives to the randomized controlled clinical trials in rare leukemias. A strategy to overcome these limitations is to develop individualized approaches “N-of-1”, based on the integration of chemical and genomics approaches developed to identify recurrent patterns of sensitivity or resistance to specific drugs [174].

As we briefly described above, T-ALL results from a multistep oncogenic transformation process that involves DNA mutations, protein expression alterations, cell cycle deregulation, anti-apoptotic mechanisms, and epigenetic modifications and their interactions with the tumor microenvironment [12,175]. DNA/RNA sequencing alone is insufficient to predict a priori response to treatment, because it does not fully recapitulate the cell-context pathway activation that can be potentially targeted. Large integrative studies on cell line panels confirm the difficulty of predicting drug responses based on only genomic data [176,177].

Furthermore, the efficacy of these new small molecules may be hampered by secondary resistance-driving relapse-associated mutations that will require drug-drug combination strategies to overcome these limitations.

An alternative approach is to develop methods to establish drug sensitivity and resistance profiling (DSRP) on primary leukemia samples to provide functional information and assist clinical decision through the identification of actionable targets. Currently, clinical studies based on DSRP are critical but anecdotal [178]. A robust and large work by Frismantas et al. tested 60 drugs on 68 ALL samples, mostly from resistant disease, in co-cultures with mesenchymal stem cells (MSCs) [179]. Each drug was tested at multiple doses and the results were analyzed with high-content imaging cell-based viability readout. Interestingly, none of the compounds affected MSCs viability, indicating a selective activity against ALL cells. Few examples of clinical responses have been reported from this study. For example, venetoclax demonstrated a potent anti-tumor activity both in vitro and in primary patient cells affected by R/R T-ALL, and delayed leukemia progression in PDX models. In addition, this study showed an unpredicted response to dasatinib in 12 T-ALL patients without the typical *ABL1* translocation. Neither recurrent genetic mutations nor altered RNA expression were linked to ABL or SRC aberrancies. Because dasatinib did not correlate with other BCR-ABL inhibitors, the author hypothesized that dasatinib inhibits SRC in T-ALL. This observation was validated in 33 adult and pediatric T-ALL samples in vitro. Subsequently, a patient with mediastinal and abdominal lymph node involvement refractory to multiple chemotherapy regimens was treated with 140 mg/die of dasatinib in monotherapy, and his disease was controlled over 5 months [179]. In a recent translational experience, we used a DSRP approach to guide clinical decision in a young woman with an early T precursor ALL, who displayed refractory disease after a standard chemotherapy program (Hyper-CVAD). T-ALL blasts showed enhanced sensitivity to venetoclax and to the proteasome inhibitor bortezomib. After internal review board (IRB) approval, our patient started a personalized therapy with the anti-BCL2 agent venetoclax (800 mg). We observed a measurable reduction in BM blasts count (from 60% to 20%) and a decrease of the mediastinum mass. Repeated measurement in T-ALL blasts with respect to sensitivity to venetoclax during treatment demonstrated a decrease in the ability to achieve a complete cytotoxic effect. To prevent the selection of resistant clones we decided to associate bortezomib (1.3 mg/m^2^ day 1,4,8,11) with the current anti-BCL2 therapy. This combination regimen was well tolerated and led to further disease reduction compatible with the enrollment of the patient in our HSCT program, as a well-matched unrelated donor was available [180].

These studies support the development of flexible chemogenomic approaches to identify personalized therapy or to modulate clinical decisions during disease progression.

## 6. Conclusions

Despite great efforts over recent decades to improve the treatment of T-ALL, almost half of children and adult patients invariably relapse. Resistance to standard chemotherapy and corticosteroids is one of the major causes of induction failure and recurrence, indicating an urgent need in understanding the underlying molecular-resistance mechanisms and exploring ways to circumvent them. The recent genomic definition of T-ALL has provided precious knowledge to develop small molecule inhibitors that specifically target relevant cellular pathways. Nevertheless, escape mechanisms are emerging even to these new drugs. Rationale and feasible combinations to enhance their activity are required to limit toxicity and improve patients’ outcome. Finally, we support the development of DSRP to identify the relevant therapeutic choice one patient at time.

## Figures and Tables

**Table 1:** Inhibitors targeting signaling pathways in preclinical and early clinical studies.

Pathway	Inhibition	Drug	Preclinical Validation		Rationale Associations to Overcome Resistance	Clinical Trial in ALL	References
			Cell-Line	Mouse Model			
IL7R	JAK1-2	ruxolitinib	×		glucocorticoid	NCT03613428 (Phase I-II, R/R ETP-ALL, + LVP)	[28,29]
PI3K/AKT/mTOR	selective PI3K	AS605240	×	×			[30]
	AKT	MK2206	×	×			[31]
	PI3K/mTOR	NVP-BEZ235 (dactolisib)	×	×		NCT01756118 (Phase I, R/R acute leukemia)	[32,33,34]
	panPI3K	NVP-BKM12 (buparlisib)	×	×		NCT01396499 (Phase I, advanced leukemia)	[35,36]
	mTOR (TORC1/TORC2)	PP242 (torkinib)	×	×			[37]
		AZD-8055	×	×			
		OSI-027	×				[38]
RAS-MAPK	MEK	CI-1040	×				[29]
NOTCH1	gamma secretase	MK-0752	×	×	glucocorticoid, JQ1	NCT00100152 (Phase I, R/R T-ALL)	[39,40]
		PF03084014	×	×		NCT00878189 (Phase I, advenced cancer and leukemia)	
		RO4929097	×	×		NCT01088763 (Phase I, R/R T-ALL and solid tumor) NCT01236586 (Phase I, pediatric R/R solid or CNS tumor, lymphoma or T-ALL)	
	SERCA	thapsigargin	×	×			[41]
		thapsigargin derivative (JQ-FT)	×	×			[42]
BRD4/MYC	BET	JQ1/CPI203	×	×	GSI, HDAC, vincristine		[43,44]
Cyclin D	CDK4/CDK6	PD-0332991(palbociclib)	×	×	glucocorticoid, everolimus	NCT02310243 (Phase I-II, MLL leukemia) NCT03132454 (Phase I, R/R leukemia) NCT03472573 (Phase I, R/R B-ALL + dexamethasone) NCT03792256 (Phase I, Relapsed pediatric ALL, + CHT) NCT03515200 (Phase I-II, R/R ALL)	[45,46,47]
		LEE001 (ribociclib)	×	×		NCT023132454 (Phase I, R/R leukemia) NCT03472573 (Phase I, R/R B-ALL) NCT03792256 (Phase I, R/R pediatric ALL) NCT02310243 (Phase II, adult MLL leukemia)	
BLC2	BH3 domain	ABT-199 (venetoclax)	×	×	MCL1 inhibitor S63845, bortezomib, PI3K inhibitor KA2237, CHT, ruxolitinib	NCT03319901 (Phase Ib, R/R ALL, + CHT) NCT03181126 (Phase I, R/R ALL, + CHT and navitoclax) NCT03194932 (Phase I, R/R AML or ambiguous lineage leukemia, + CHT) NCT03808610 (Phase I, R/R B or T-ALL, + low intensity CHT) NCT03504644 (Phase I, R/R B or TALL, + vincristine liposomal)	[48,49,50,51,52,53,54,55,56]
Nucleo-cytospasmic traffiking	Nuclear export (SINE)	KPT-330 (selinexor)	×	×	CHT	NCT022112561 (Phase I, R/R leukemia or MDS, + fludaradine and cytarabine) NCT02091245 (Phase I, R/R pediatric ALL and AML)	[57,58,59]

SERCA: sarco/endoplasmic reticulum calcium ATPase. BET: BRD extra terminal. SINE: slective inhibitor of nuclear export. GSI: gamma secretase inhibitors. HDAC: histone deacetylase. CHT: chemotherapy. R/R: relapsed/refractory. AML: acute myeloid leukemia. ALL: acute lymphoblastic leukemia. MDS: myelodysplastic syndromes. ETP: early-T precursor. LVP: Levo-asparaginase, vincristine, prednisone. CNS: central nervous system.
